# Milled in versus round bar for mini implant retained mandibular overdentures: a 8-year retrospective radiographic study of peri-implant bone changes and posterior ridge resorption

**DOI:** 10.1186/s12903-025-06689-6

**Published:** 2025-08-16

**Authors:** Nermeen El Sayed El-Khamisy, Radwa Mohsen Kamal Emera, Heba Nabil Awad

**Affiliations:** 1 Department of Prosthodontics, Mansoura University, Mansoura, Egypt; 2 Faculty of Dentistry, Mansoura University, Mansoura, Egypt

**Keywords:** Implant-supported overdenture (IODs), Round joint bar, Milled bar, Marginal bone loss, Posterior ridge resorption

## Abstract

**Objective:**

This retrospective radiography study’s main objective was to compare round and milled bars for mandibular overdentures assisted by 4 mini-dental implants (MDI) on peri-implant marginal bone loss (MBL)and posterior mandibular ridge resorption(PRR) after 8 years.

**Materials and methods:**

Thirty male Participants in a retrospective analysis were treated between 2016 and 2024 who had four interforaminal mini implants supporting an overdenture on a milled bar. Two equal groups of 15 patients each were randomly assigned to receive mandibular overdentures: Group I received overdentures retained by milled bars, while Group II received overdentures retained by round joint bars. After an 8-year follow-up period, a comparison between the two bar designs attachment used for IODs retention. The evaluation of the posterior ridge resorption (PRR) and peri-implant marginal bone loss(MBL) was done after three years (T3), five years(T5), and eight years later (T8)after insertion. The data was analyzed using the Statistical Package of Social Science (SPSS) program for Windows (Standard version 24).

**Results:**

Compared to Group II, Group I’s MBL was noticeably higher. As time went on, MBL rose noticeably for both groups. At certain intervals, Group II’s PRR was significantly higher than its Group I.

**Conclusions:**

The design of the anchorage system has a major impact on posterior ridge resorption and peri-implant bone loss when four mini-implants are utilized to anchor mandibular overdentures.

**Clinical relevance:**

As mini implants are often seen as a cost-effective solution for patients with limited bone, it is important to understand their long-term performance to make more informed decisions in clinical practice.

**Clinical trial registry number:**

(NCT06185283). Retrospectively registered on (29-12-2023).

## Introduction

The accepted treatment for a mandible that is completely edentulous is implant-retained overdentures. The use of dental implants to support complete mandibular dentures is an accepted and effective treatment, achieving a success rate of over 95.5% [1]. These prostheses are known as implant-supported overdentures (IODs) when they rely entirely on implants or implant-assisted overdentures when partially supported by implants and soft tissues [2]. Compared to fixed restorations, overdentures offer advantages such as lower costs, facial tissue support, and reduced need for bone grafting [3]. Supporting overdentures with four implants provides better stability, reduces posterior bone resorption, and minimizes mucosal support dependency compared to two-implant overdentures [4].

Reduced-diameter implants, commonly referred to as mini dental implants (MDIs), with diameters ranging from 1.8 to 3.2 mm, are used in cases with limited bone anatomy. They are cost-effective, offer quicker recovery and fewer complications, are minimally invasive, and have a success rate between 83.9% and 97.5% [5–8]. They are particularly beneficial for elderly patients due to their conservative nature and positive impact on quality of life [6, 7]. IODs are retained using attachments, which are mechanical tools used to stabilize, fix, and retain a prosthesis in place. The design of the attachments being employed has a major impact on the load transmission to implants [9]. Clinically, attachments that allow for an even distribution of occlusal stresses between abutments are preferred, concerning implant longevity and preserving the bone [10]. Attachments can be splinted (bar attachments) or non-splinted. Splinted attachments distribute stress more effectively, reducing peri-implant bone loss [11, 12]. Bar attachments provide implant splinting, stress distribution, and stability in atrophic ridges [13–16]. There are two bar design concepts: round bar joints and milled bars that can be used to retain complete overdentures with splinting implants. In addition to their stress-relieving properties, bar joint attachments permit the overdenture to move somewhat vertically and rotationally [17]. Conversely, precisely milled, vertically parallel guiding planes in milled bars enable close contact with the denture base, providing increased stability and resistance against lateral and rotational forces [18]. The degree to which the connecting bar permits the implant-assisted overdenture to move both vertically and rotationally determines the amount of stress transmission to the posterior remaining alveolar ridge [19, 20].

The peri-implant alveolar bone at the implant crystal region is a crucial factor in determining the health of the implant [21]. Radiographic interpretation is a common method to evaluate the available bone height changes [22]. The primary factor influencing a successful treatment outcome is stable marginal bone levels surrounding implants [23, 24]. The majority of IOD support is provided by the implants and the mucosal layer of the alveolar ridge [25]. Numerous studies have looked at how IODs affect time-dependent posterior mandibular ridge resorption (PMRR), yet there is a risk that rotating the IODs around the implants will increase the occlusal load on the posterior mandibular ridge [26–28]. A frequent screening technique for patients who are dentate and edentulous in big institutional practices is panoramic radiography. According to several studies [29–31], traced panoramic radiographs can be used to measure bone resorption in the mandible and maxilla. They described a technique for dividing the mandible into two regions: the alveolar ridge’s extent determines one region, and anatomical landmarks resistant to resorption determine the other. The term “area index” refers to the proportional value between the two areas.

The goal of the present study was to retrospectively evaluate how two distinct bar designs would affect the MBL and PRR in complete mandibular overdentures when it came to splinting mini-implants. after the first three (T3), after five years (T5), and after eight years (T8) from the time of overdenture insertion.

The study hypothesized that for four mini-implant retained overdentures on MBL and PRR, there would be no discernible difference between round joint and milled bar attachments.

## Methods

### Study design

**Fig. 1:**
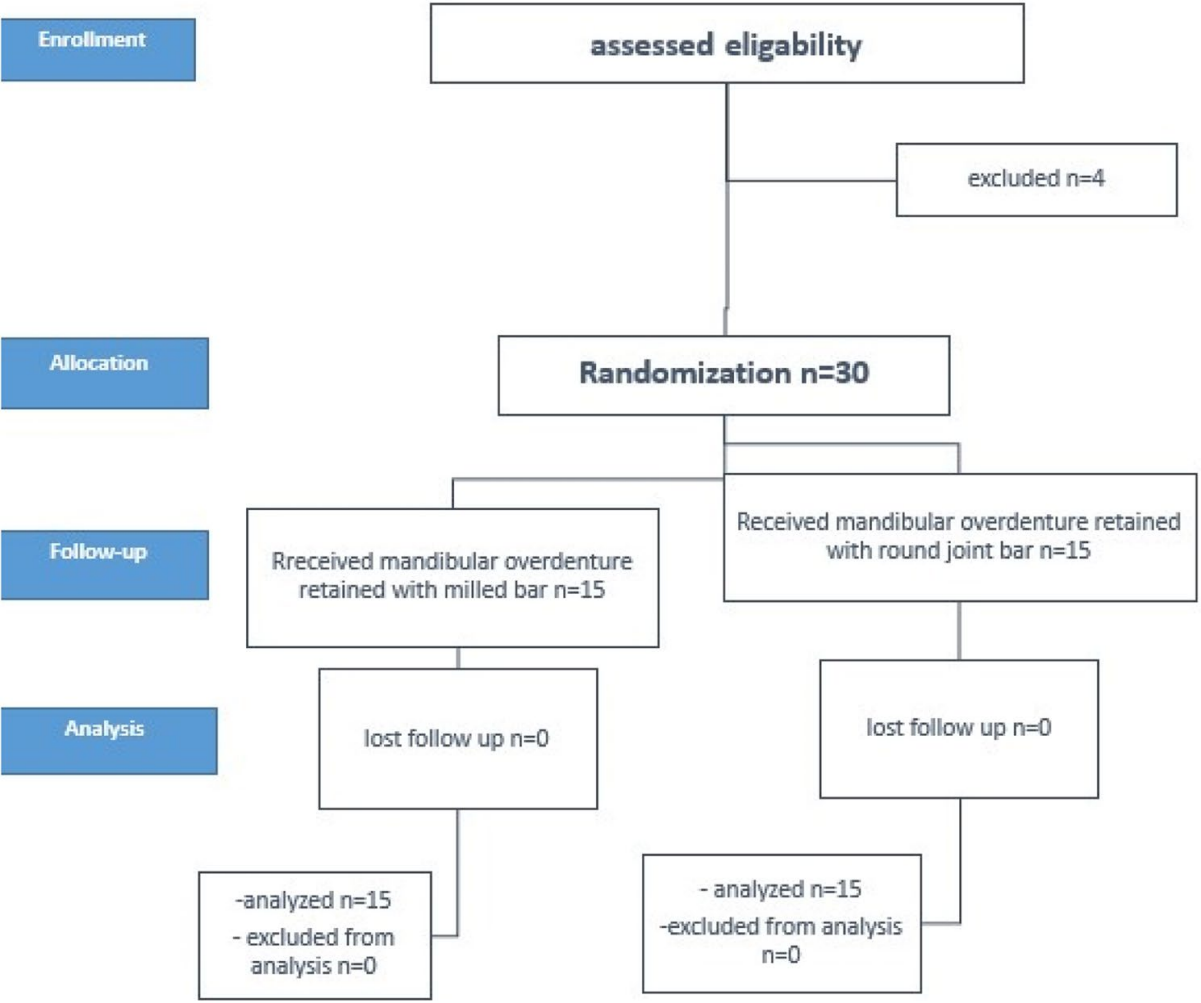
The study procedures’ flowchart

An 80% power sample size was inspected for effectiveness, depending on an earlier investigation [32]. The G*Power software (version 3.1.5, Kiel, Germany) was used to do the power analysis. Thirty-four male patients with mandibular overdenture assisted by four mini-implants opposed by conventional maxillary denture were chosen for this retrospective study, aged from 50 to 65 years (mean 57.5 years), and were involved in this clinical trial between December 2015 and December 2023. Thirty of them completed this clinical study, and 4 patients did not complete the follow-up periods. The patients were randomly and equally divided into two groups (*n* = 15 per group), utilizing arbitrary numbers produced by an Excel spreadsheet. Each group consisted of 15 male patients, as indicated by the bar design used for retention of the IOD. Group I: patients received lower IOD retained by milled bars, and Group II: patients received lower IOD retained by round joint bars. The study procedures’ flowchart is displayed in Fig. 1. The study’s procedures were carried out at the prosthodontic department. The Declaration of Helsinki’s ethical guidelines were adhered to in this study. The Faculty of Dentistry ethics board committee approved this clinical investigation, and it was given the ethical approval number (M01010023RP). The study’s protocol was retrospectively registered in clinicaltrials.gov (No. NCT06185283). Following an explanation of the study procedures, every patient signed the consent form. This clinical study reports on the radiographic results of a clinical trial that compares the mandibular over-denture retained by different bar designs on four mini implants and a maxillary complete denture in edentulous patients on MBL and PRR.

### Data collection

All of the patients who were chosen for this study were fully edentulous and satisfied the following requirements: patients were completely edentulous with mandibular residual ridge (5 mm of bone width and minimum 17 mm of bone height covered with healthy firm keratinized mucosa with at least 2 mm thickness) in the inter-foraminal region which were determined by Cone Beam CT to support an implant length of at least 10 mm; and a ridge width of 5 mm which is thought to be the minimal width to take flapless procedure error into account. They also had edentulous mandible for a minimum of six months, and the causes of edentulism were trauma, aging, and carious lesions of teeth, with no acute or chronic periodontitis. Patients had class I maxillomandibular relations, which are predicated on tentative jaw relations, and had 12–15 mm of restorative space from the crest of the ridge to the incisal edge, which is usually required with the bar system. This distance consists of 4–6 mm for the bar system, at least 1 mm for the space between the inferior surface of the bar and the ridge, and 6–8 mm for the teeth, the acrylic denture base, and the clip. They had good motivation to follow time-based recalls.

Exclusion criteria were any systemic disorders that could impede the osseointegration of implants, the patients were excluded, people having a history of parafunctional behaviors like clenching or bruxism, people who showed symptoms of untreated temporomandibular disorders or those with bone metabolic abnormalities; people with uncontrolled systemic or oral conditions that required additional treatment; were not included in the study.

Patients were classified equally and randomly into two groups (*n* = 15 per group) using arbitrary numbers created in an Excel spreadsheet. Group I patients were given mandibular (IODs) retained by a milled bar opposed by a conventional maxillary complete denture. For Group II patients were recessive mandibular (IODs) retained by round joint bar opposed by conventional maxillary complete denture. The patients were assigned to treatment groups and randomly assigned by dental experts who were not aware of the treatment groups.

For both groups, 1- Mandibular and maxillary master casts were made using secondary impressions of rubber base material (Coltenespeedex, Switzerland). The maxillary cast and the mandibular duplicate were used to create record blocks for the maxillomandibular relation record. When setting the artificial teeth made of acrylic resin, the balanced lingualized occlusion technique was used. Dentures were flasked, finished, and polished. The mandibular final overdenture and maxillary complete denture.

### Surgical procedures

Surgical procedures were conducted by the oral surgeon under local anesthesia, all implants were placed using a one-stage flapless implant placement procedure in the intraforminal area in the mandible. Following the surgical guide template’s entire seating, 3 mm circular soft tissues matching each MDI position were cut using a tissue punch. Under heavy irrigation, the pilot drill (1.1 mm diameter) was used to initially drill the four implants, followed by 2nd drill (2 mm diameter). Ultimately, the single piece MDI of 2.9 mm diameter and 15 mm length (3 M™, ESPE™ MDI, USA) was torqued to its final position at 35–45 N/cm using a torque ratchet wrench. The inter-implant distances should be wide enough to accommodate the bar and the clips to avoid distortion and unsatisfactory retention of at least 4 mm between each implant. A tissue conditioning substance was applied after the mandibular denture fitting surface was alleviated.

### Prosthetic procedures

One week following implant placement, intraoral scanning using the 3 SHAPE TRIOS Scanner was performed to capture the implant positions.

### Group I: milled bar construction

For Group I, a milled bar construction was designed with the 3Shape Dental Designer. Four nearly identical abutments, each 2 mm thick, were placed over the implant abutments. A parallel-wall bar design (Rehin 83) was selected and designed to fit over the four implant abutments.

### Group II: round-bar joint construction

**Fig. 2:**
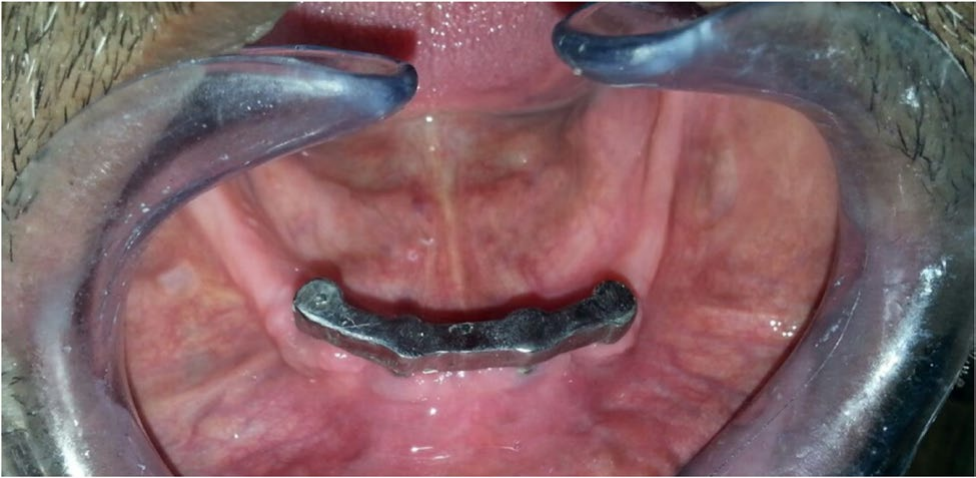
Milled in bar cemented intra-orally on 4 intraforaminal mini implants

**Fig. 3:**
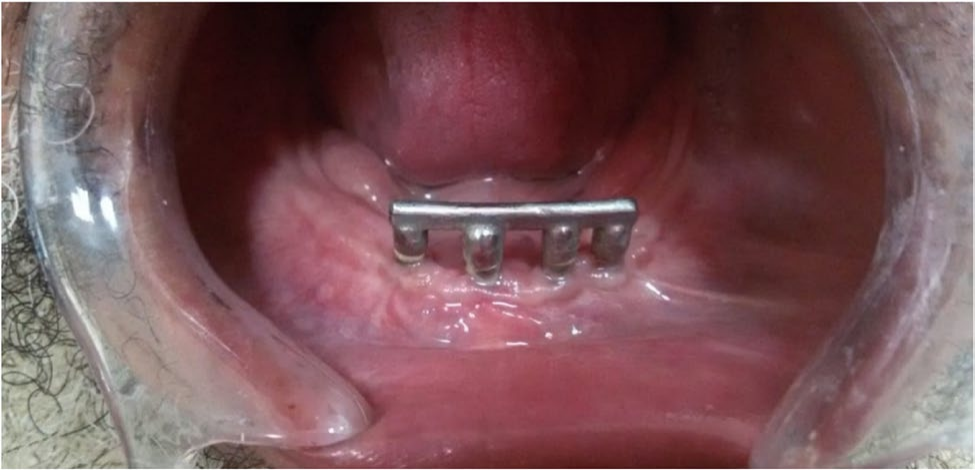
Round bar cemented intra-orally on 4 intraforaminal mini implants

For Group II, a round-bar joint construction approach was used. Four nearly identical abutments (2 mm thick) were placed over each implant abutment. The selected Rehin bar (RHIN 83 OT BAR multi-use) was overlapped onto the four virtual abutments to form the final bar design. Subsequently, the STL files of both designs were exported to a milling machine for the fabrication of the resin try-in. Following the resin try-in, the resin bars were invested to prepare for the metal bars. The final metal bars (milled Fig. 2 and joint round bars Fig. 3) were then tried intra-orally to ensure passive fit before proceeding with cementation.

Retentive clips pick-up procedure: The bars were set in place two weeks after the procedure. Denture insertion and removal were done repeatedfly while using pressure-indicating paste in an attempt to achieve a passive fit for the mandibular overdenture. Next, under a small closure force applied by the patient in centric occlusion, three retentive custom-made metal clips (for milled bar) and ready-made plastic clips (for round bar) were picked up in the mandibular overdenture fitting surface. The patients were scheduled for standard follow-up appointments and given instructions on good oral hygiene.

### Radiographic evaluation

**Fig. 4:**
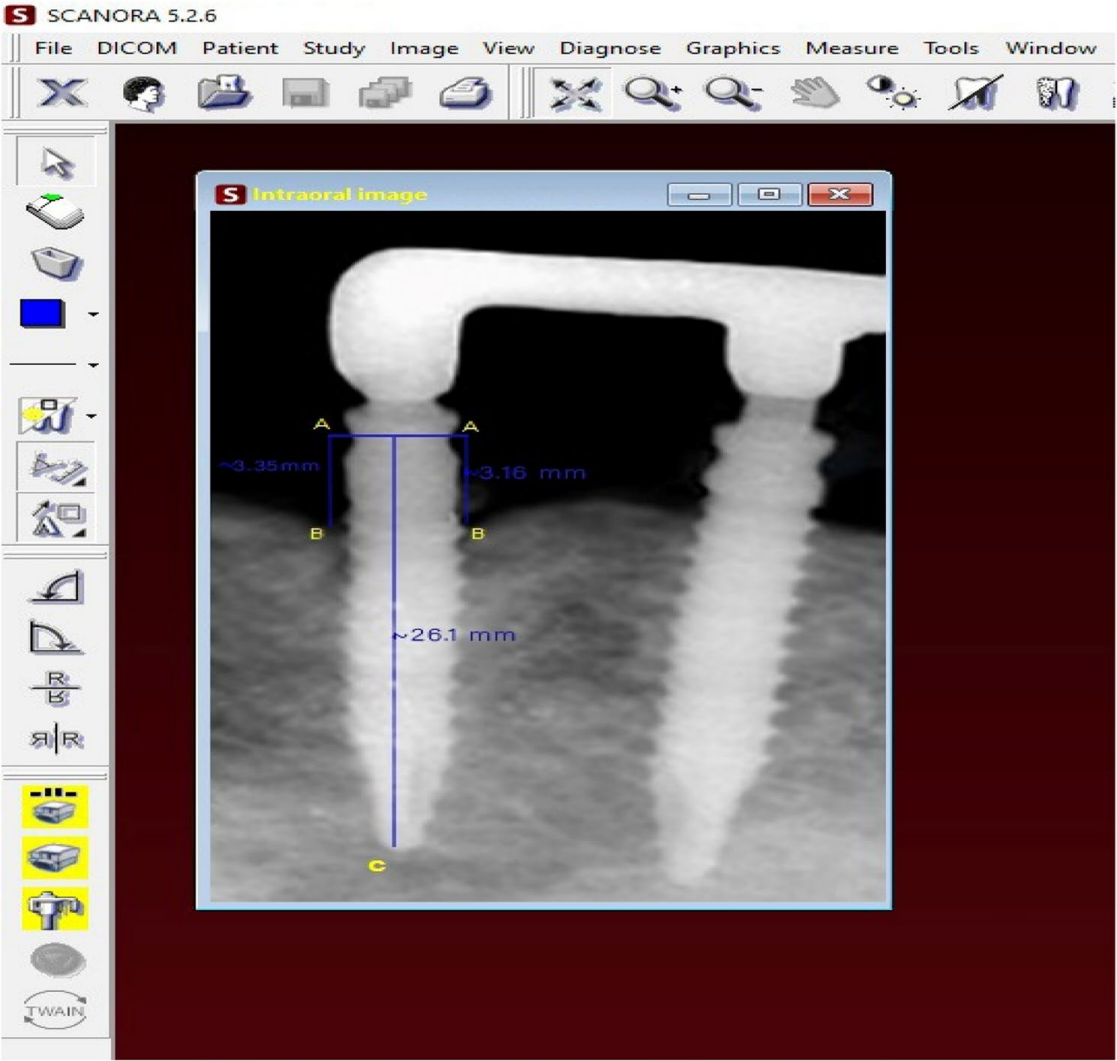
Measurements of the peri-implant vertical bone loss. Measuring implant length in the radiograph. b-Bone height measurement in millimetres as the distance from point A (the implant neck) to point B (the most coronal point of bone-implant contact)

**Fig. 5:**
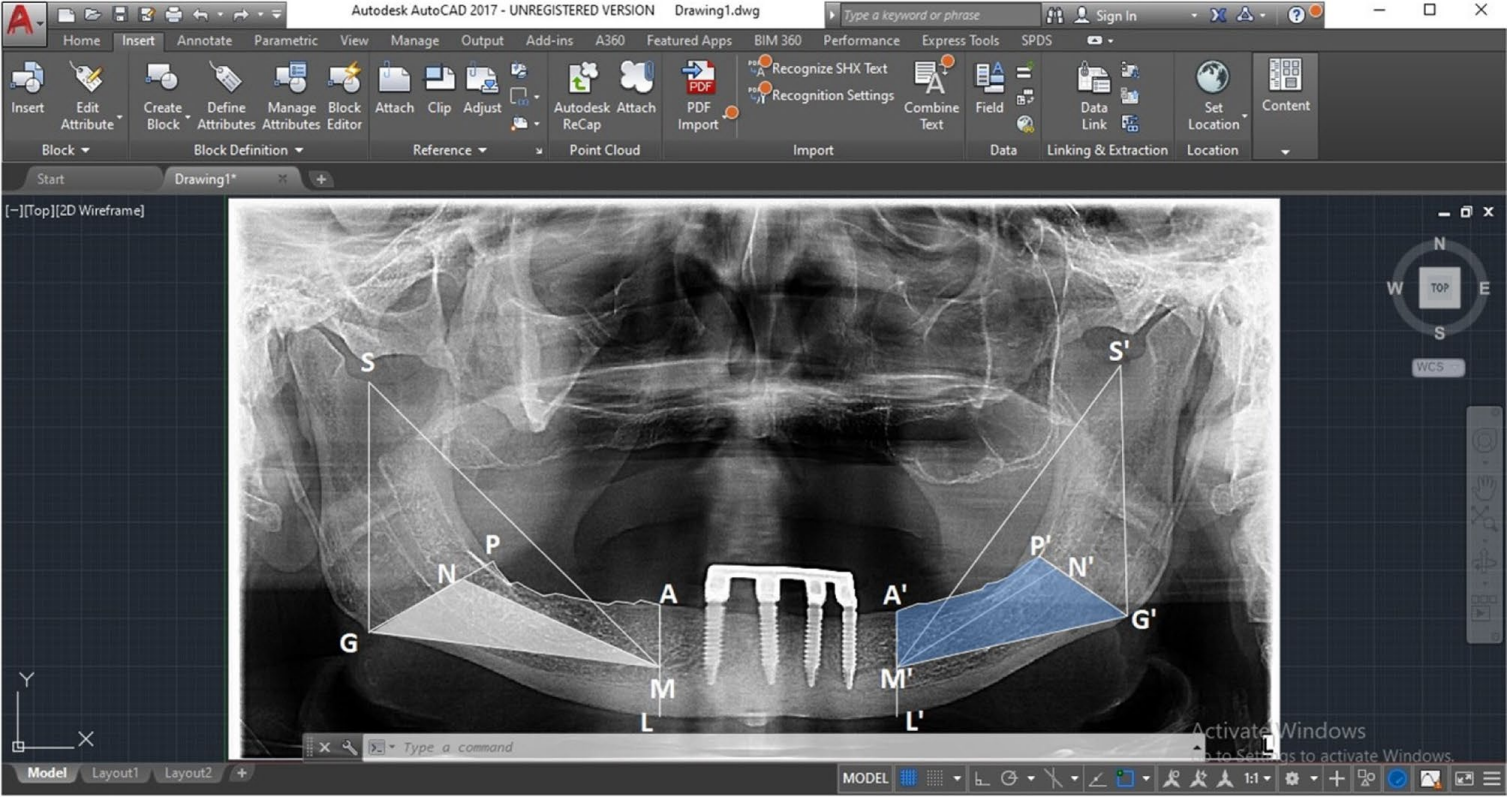
Measurements of posterior alveolar ridge resorption. were performed using the assisted drawing program AutoCAD 2017 (Autodesk)

- For MBL evaluation, standardized intra-oral radiographs using the long cone paralleling technique were produced by (DIGORA Optime Instrument; SOREDEX, Finland) of each implant. The MBL was assessed radiographically after the first three (T3), after five years (T5), and after eight years (T8) from the time of overdenture insertion. Dental Digital Film (Phosphor IP Plates) was scanned. The radiographic images were traced by using SCANORA Lite software 3.2.6 (PaloDex Group, Finland) to calculate magnification errors. To compute magnification error and determine the true values of peri-implant bone measures, the length of the implant on the radiograph (point C) and the real implant length were compared. The distance between point A (the implant neck) and point B (the most coronal point of bone-implant contact) was used to calculate bone height in millimeters Fig. 4. Bone levels were subtracted at the first three, five, and eight years from the time of insertion to calculate MBL. MBL was computed at the proximal surfaces of each implant, and mean values were computed for every patient.

- For PRR evaluation, panoramic radiographs were acquired for each patient. To standardize all photos, the panoramic unit (CRANEX 3DX Instrument; SOREDEX, Finland) was operated at 70 kV with a steady current of 10 mA and an exposure duration of 16.4 at three, five, and eight years to evaluate PRR. A proportional measuring method like that described by Wilding et al. [31] was used to assess the bilateral posterior regions of the remaining ridges on the panoramic radiographs. Triangles on the right (M-S-G) and left (M’-S’-G’) sides were constructed using a line that connected M (lower border of mental foramen), S (sigmoid notch), and G (gonion) to track the posterior ridge resorption (PRR) (experimental region). The posterior alveolar ridge area represented by the radiographic PAMG area is measured using computer software (AUTOCAD^®^ 17) Fig. 5. It was divided to the MNG area, which is based on relatively fixed bony landmarks. The ratio between the two is unaffected by radiographic magnification, and the mean value of both sides is used to calculate the PRR for each subject. By subtracting the ratio at the time of insertion from the ratio at 3 years (T3), subtracting the ratio at 5 years from the time of insertion (T5), and subtracting the ratio at 8 years from the time of insertion (T8). For every group, the difference in ratio in PRR was computed. The same examiner collected the data.

### Statistical method

The data was analyzed using the Statistical Package of Social Sciences (SPSS) program for Windows (Standard version 24). First, the data’s normality was assessed using the Shapiro-Wilk test. Continuous variables were displayed as mean ± SD (standard deviation) for regularly distributed data. The independent t-test was used to compare the two groups, and the repeated measured ANOVA test, which was followed by a repeated paired t-test, was used to compare more than two durations in the same group. The significance threshold for each of the aforementioned statistical tests is set at the 5% level. When *p* < 0.05, the results were deemed significant. The results are more significant if the *p*-value is less.

## Results

Thirty-four male patients with a mean age of 57.5 years were included in the study, and thirty of them completed this clinical study with a dropout rate of 12%. The comparisons of MBL within groups and between different observation times are presented in **(**Table 1**)**. Increased bone loss was observed overtime with a tendency Group I recorded a significantly higher MBL than Group II at T3, T5, T8 (independent t test *p* < 0.001), and there was a statistically significant difference at different times of evaluation within groups where (Repeated measure ANOVA test, *P* < 0.0001), followed by repeated paired test between groups there was significantly difference between (T3, T5),(T5, T8) and (T3, T8) where *P* < 0.0001. The mean marginal bone loss was 1.28 ± 0.02 mm for group I and 1.08 ± 0.06 mm for group II during the first 3 years (T3). After 5 years (T5), the mean was 1.68 ± 0.03 mm for group I and 1.45 ± 0.05 mm for group II. After 8 years (T8), the mean was 1.90 ± 0.04 mm for group I and 1.72 ± 0.05 mm for group II, as shown in Table 1.

**Table 1:** Comparison of MBL at different follow-up periods between groups and within each group

	Group I (*n* = 15) Mean ± SD	Group II (*n* = 15) Mean ± SD	Test of significance*, *p*-value
MBL (T3)	1.28 ± 0.02	1.08 ± 0.06	t = 11.468 *p* < 0.001
MBL (T5)	1.68 ± 0.03	1.45 ± 0.05	t = 14.803 *p* < 0.001
MBL (T8)	1.90 ± 0.04	1.72 ± 0.05	t = 11.838 *p* < 0.001
Test of significance#, *p*-value	F = 2016.279, *p* < 0.001	F = 720.890, *p* < 0.001	
MBL (T3) – (T5)	t = − 56.941, *p* < 0.001	t = − 19.846, *p* < 0.001	
MBL (T3) – (T8)	t = − 70.220, *p* < 0.001	t = − 31.854, *p* < 0.001	
MBL (T5) – (T8)	t = − 17.089, *p* < 0.001	t = − 25.688, *p* < 0.001	

*Independent t-test, #: Repeated measure ANOVA followed by repeated paired t-test, Group I: Milled bar, Group II: Round bar joint.T3: first 3 years after insertion, T5 after 5 years of insertion, T8 after 8 years of insertion

The comparison of posterior ridge resorption (PRR) between both groups and within each group is presented in Table 2.

**Table 2:** Comparison of PRR at different follow-up periods between groups and within each group

	Group I (*n* = 15) Mean ± SD	Group II (*n* = 15) Mean ± SD	Test of significance*, *p*-value
PRR (T3)	1.30 ± 0.03	1.43 ± 0.03	t = − 11.297 *p* < 0.001
PRR (T5)	1.61 ± 0.03	1.79 ± 0.08	t = − 8.350 *p* < 0.001
PRR (T8)	1.79 ± 0.04	2.05 ± 0.11	t = − 8.833 *p* < 0.001
Test of significance#, *p*-value	F = 1047.294, *p* < 0.001	F = 427.171, *p* < 0.001	
PRR (T3) – (T5)	t = − 34.252, *p* < 0.001	t = − 17.916, *p* < 0.001	
PRR (T3) – (T8)	t = − 43.460, *p* < 0.001	t = − 22.016, *p* < 0.001	
PRR (T5) – (T8)	t = − 14.798, *p* < 0.001	t = − 20.173, *p* < 0.001	

*Independent t-test; #: Repeated measure ANOVA followed by repeated paired t-test. Group I: Milled bar, Group II: Round bar joint, T3: first 3 years after insertion, T5: after 5 years of insertion, T8: after 8 years of insertion

The comparison of PRR at different times between groups and within each group is shown in (Table 2). Throughout the assessment period, there were notable variations between the groups at T3, T5, and T8 (Independent t-test, *p* < 0.001), and there was a statistically significant difference at different times of evaluation within groups (Repeated measure ANOVA test, *P* < 0.0001). Group II recorded higher PRR values over the observation period as compared to group (I) The mean PRR was 1.30 ± 0.03 mm for group I and 1.43 ± 0.03 mm for group II during the first 3 years (T3). After five years (T5), the mean was 1.61 ± 0.03 mm for group I and 1.79 ± 0.08 mm for group (II) After 8 years (T8), the mean was 1.79 ± 0.04 mm for group I and 2.05 ± 0.11 mm for group II, as shown in Table 2.

## Discussion

In the case of four-mini implant-assisted overdentures, there is not enough data to conclusively say whether the properties of bars affect posterior ridge resorption and peri-implant marginal bone loss. The present study aimed to evaluate changes in peri-implant marginal bone loss and posterior mandibular ridge resorption in patients with mandibular mini-implant overdentures, comparing two groups using different bar designs for splinting the mini implants. The two bar designs included a milled bar, considered a rigid anchoring system, and a round joint bar, functioning as a resilient anchoring system. A flapless surgical technique was used to place mini implants to preserve the blood supply, minimize damage to both the endosteal and periosteal bone, and maintain the height of the peri-implant bone post-surgery [33, 34].

Dental implants supported by a bar (also referred to as bar-supported implants) are associated with higher success rates compared to individual implants due to several factors. In the present study, four intra-foraminal Mini Dental Implants (MDI) were utilized, with a 100% survival rate observed in both groups throughout the observation period. One of the primary benefits of a bar-supported implant system is its capacity to more effectively distribute occlusal (biting) forces across multiple implants. The bar functions as a rigid, stable support for the prosthesis, thereby enhancing the overall stability of the dental restoration. By distributing the functional load across multiple implants, the bar splint reduces the risk of failure for any single implant, consequently improving the overall success rate of the implant system [35].

A retrospective study was carried out on the patients who received implant-assisted lower overdentures with two distinct bar designs. The long-term failure of implants is frequently linked to partial marginal bone loss [36, 37]. Consequently, measuring peri-implant marginal bone loss is a critical outcome parameter in the field of dental implants, serving as an important sign of implant success. When the magnitude of the applied load exceeds the physiological threshold for bone adaptation, bone-implant anchorage may be compromised, leading to a potential loss of implant stability and, ultimately, a reduction in the overall success of the implant [38].

In the current investigation, the mean bone level (MBL) for the milled and joint bar groups was 1.28 ± 0.02 and 1.08 ± 0.06, respectively, three years after loading. During the first three years of function, MBL at the peri-implant surfaces was generally considered within an acceptable range of 1 to 2 mm. Other publications have reported different MBL values of 1.5, 1.8, and 2.2 mm [39–41]. It was recently discovered that the MBL range after one year of function falls between 0.13 ± 0.35 mm and 1.03 ± 0.65 mm [42–44]. In the present investigation, the two groups recorded annual bone loss, though it was less than 0.2 mm. The average bone level surrounding the implants actually stayed constant throughout 5 to 8 years. The highest MBL mean values of both groups were observed in the 8 years. Reduced mini-implant diameter has been shown to enhance stress concentration at the bone/implant contact, which may be the reason for the significant bone loss observed at T8 compared to T3 and T5 for both groups.

Compared to group II (round bar joint), MBL was noticeably greater in group I (milled bar) after a long evaluation period at T3, T5, and T8. This could be a result of the extended assessment time, making it possible to identify marginal bone alterations. This result may be due to the rigidity of the milled bar, precision fit, and the way they transfer concentrated forces to the implants, especially around the marginal areas where bone resorption can occur more easily [45].

Resilient joint bars allow the overdenture base to rotate in response to occlusal pressure, supporting both the implants and the mucosa [46]. Peri-implant bone strain was significantly reduced when round bars were used [47]. On the other hand, the prosthesis cannot rotate when rigid milled bars are used and transmit all occlusal stresses to MDIs [48, 49]. This explains why this group was recorded as having a higher MBL than the round joint bar group. Stability and resistance to rotational and lateral forces are provided by precisely fitting the overdenture base to milled bars, which is accomplished by precise milling [49–51]. The weight can be distributed among the implants with rigid bar attachments, preventing the posterior residual ridges from being shared [51]. Due to unrestricted overdenture rotation during function, this treatment method also causes ongoing bone resorption in the posterior region [52–54]. Nevertheless, adding more implants improves retention, reduces bone loss, and improves stress distribution to improve treatment results [55].

Posterior bone resorption in bar-supported overdentures occurs due to several factors, including the uneven distribution of chewing forces, inadequate implant support in the posterior areas, lack of proper bone stimulation, and excessive stress on the ridge. The posterior part of the jaw is often more vulnerable due to factors like lower bone density and fewer implants, leading to higher stress in these areas. To prevent posterior bone resorption, it is important to ensure optimal implant placement, balanced force distribution, and proper fitting and design of the bar-supported overdenture system [35, 56].

A related study that assessed the resorption of posterior ridges caused by complete lower overdentures held in place by 4 splinted MDI with round and milled bars similarly demonstrated the biomechanics characteristics of these two distinct bar types. In comparison to round bar joints, it was determined that milled bars seemed to spread the load on dental implants and prevent lateral and rotational movements. Milled bar overdenture movement is affected only by the path of removal that is beneficial for avoiding prosthesis upkeep and associated with decreased resorption of mandibular posterior ridges [57–59]. Round joint bars, on the other hand, contribute to greater posterior ridge resorption because their flexibility and less precise force distribution can result in more concentrated stress in the posterior jaw, where bone is already more vulnerable due to lower density [60]. Also, a resilient anchorage system (round joint bar) that allows vertical and rotary movements of the overdenture. So, we increase the prosthesis tissue support during function [35]. Round bars provide combined mucosa-implant support, allowing the prosthesis to freely rotate vertically while in use and transferring a range of loads to the posterior edentulous region with minimal load on the implants [61–63]. The result of the current investigation is explained by this biomechanical behavior. The round bar group recorded higher PRR over the follow-up period than the milled bar group.

The null hypothesis was thus rejected, according to which there are variations between the two bar designs on marginal bone loss and restorative changes in the posterior mandibular ridge.

The decision between a round bar and a milled bar ultimately comes down to several criteria, including the patient’s bone quality, implant placement, occlusal forces, and treatment objectives. The durability of the overdenture system can be ensured, and the hazards of bone resorption reduced with careful planning and construction.

One limitation of this study was the small sample size. Additionally, only two bar designs were evaluated, limiting the scope of comparison. Further research incorporating a larger sample size and additional bar attachment designs is necessary to validate these findings and explore potential variations in outcomes.

## Conclusions

The use of four intra-foraminal mini-implants with bar attachments for mandibular complete overdenture retention over eight years presents a biologically viable alternative to the conventional approach of two standard-sized implants.

For optimal peri-implant bone preservation, a round bar attachment is recommended, whereas a milled-in bar design may be more effective in minimizing posterior ridge resorption.

## Data Availability

All data are available upon request from the corresponding author.
